# Fusion vs. Isolation: Evaluating the Performance of Multi-Sensor Integration for Meat Spoilage Prediction

**DOI:** 10.3390/foods14091613

**Published:** 2025-05-02

**Authors:** Samuel Heffer, Maria Anastasiadi, George-John Nychas, Fady Mohareb

**Affiliations:** 1 Bioinformatics Group, Faculty of Engineering and Applied Sciences, Cranfield University, Cranfield MK43 0AL, Bedfordshire, UK; s.heffer@cranfield.ac.uk (S.H.); m.anastasiadi@cranfield.ac.uk (M.A.); 2 Agricultural University of Athens, 11855 Athens, Greece; gjn@aua.gr; 3 International Joint Research Lab (China and Greece) of Digital Transformation as an Enabler for Food Safety and Sustainability, Tai’an 271018, China

**Keywords:** spoilage, machine learning

## Abstract

High-throughput and portable sensor technologies are increasingly used in food production/distribution tasks as rapid and non-invasive platforms offering real-time or near real-time monitoring of quality and safety. These are often coupled with analytical techniques, including machine learning, for the estimation of sample quality and safety through monitoring of key physical attributes. However, the developed predictive models often show varying degrees of accuracy, depending on food type, storage conditions, sensor platform, and sample sizes. This work explores various fusion approaches for potential predictive enhancement, through the summation of information gathered from different observational spaces: infrared spectroscopy is supplemented with multispectral imaging for the prediction of chicken and beef spoilage through the estimation of bacterial counts in differing environmental conditions. For most circumstances, at least one of the fusion methodologies outperformed single-sensor models in prediction accuracy. Improvement in aerobic, vacuum, and mixed aerobic/vacuum chicken spoilage scenarios was observed, with performance enhanced by up to 15%. The improved cross-batch performance of these models proves an enhanced model robustness using the presented multi-sensor fusion approach. The batch-based results were corroborated with a repeated nested cross-validation approach, to give an out-of-sample generalised view of model performance across the whole dataset. Overall, this work suggests potential avenues for performance improvements in real-world, minimally invasive food monitoring scenarios.

## 1. Introduction

Meat is an important staple in diets worldwide, containing a large spread of macro- and micro-nutrients. Poultry and beef are important livestock for global markets, accounting for ~33% and ~24% of consumed meat, respectively, according to the United Nations Food and Agriculture Organisation [1]. Despite its nutritional importance and high demand, fresh meat is highly perishable, which limits its shelf life and marketability. This perishability primarily arises from the activity of endogenous enzymes and microbial growth, notably Pseudomonas spp. under aerobic storage conditions and Lactic Acid Bacteria (LAB) under modified atmosphere packaging. Concerning the latter, their activity is linked to many of the undesirable outcomes that characterise poor-quality meat such as unpleasant flavours, malodour, or changes to visual and textural appearance. While stale or spoiled food is not necessarily unsafe, the negative impact of spoilage-causing microbial activity negatively impacts consumer’s acceptance and necessitates steps to control or even inhibit the microbial population growth. Amongst others, refrigeration and the use of modified atmosphere packaging (MAP), which may include the application of vacuum conditions or ratios of gases incompatible with targeted microbial growth for that specific food type, including nitrogen, carbon dioxide, or oxygen, have been applied [2,3].

Improving the accuracy and responsiveness of food quality assessment has been a topic of active research for many years, partly driven by the growing interest in improving the state of food supply infrastructure [4]. Furthermore, clear monetary motivations exist for producers and suppliers to optimise their operations through minimisation of wastage—globally, an estimated 13% of food is wasted or lost between harvest/production and retail [5]. Food testing methodologies have been standardised within legislation at a national and international level (EFSA 2073/2005) [6]. Current strategies feature laboratory-based methods for quantifying the sizes and types of microbial populations within food samples [7], in addition to organoleptic and sensory analyses [8,9], which together form the foundation of food quality monitoring.

Over the last 10–15 years, data-driven approaches for employing sensor technologies to digitise the physico-chemical properties of food samples in a non-/minimally invasive, high-throughput manner have been employed [4,10,11]. Application of sensor technologies may serve to increase the responsiveness and coverage of testing within the food production and distribution pipeline and complement the capabilities of the aforementioned methodologies, whilst not suffering the shortcomings of conventional techniques, i.e., their time-consuming, technically involved nature that requires specialist personnel [4].

The motivations for the deployment of these sensor types throughout the production and distribution pipeline are summarised in the concept of process analytical technology (PAT) [7]. A plurality of sensor modalities exists, including biomimetic sensors that are designed to record a sample’s chemometric properties in a manner that emulates the human sensory experience (e.g., electronic nose and electronic tongue), and spectroscopic sensors that record the optical properties of a sample [4].

Techniques such as Fourier transform infrared (FTIR) spectroscopy, Raman spectroscopy, and multispectral imaging (MSI) have been investigated in meat quality and spoilage analyses [12,13,14]. A large number of machine learning algorithms, such as partial least squares discriminant analysis (PLS-DA) and regression (PLS-R) models, have been used to analyse such data [4]. Spyrelli et al. compared microbial count regression performance to models built on sample images captured with the Videometer proprietary MSI platform and confirmed the utility of FTIR spectroscopy to detect spoilage in chicken [14]. The researchers found that while FTIR-trained models outperformed MSI models, the difference may have been due to intra-batch variation rather than the Videometer data having less predictive power. The authors also use MSI/Videometer sensor data to predict chicken slaughter time [15]. In their machine learning objectives, the resultant models used data from one sensor modality as predictor variables. While such machine learning approaches provide a promising prediction accuracy, the combination of the sensor platform and the applied machine learning algorithm plays a major role in the achieved RMSE value. Therefore, sensor/data fusion may improve machine learning model prediction if different sensor modalities operate in different observational spaces and record different sample states, which is the main hypothesis we aim to test within the context of this work.

Data fusion is a wide topic within the information–technology domain which covers many approaches for combining useful information originating from different sources. Within the bioinformatics sphere, the main approaches for reaching these objectives can be subdivided into the following categories, based on the stage of the information processing workflow at which the fusion operation takes place:

Early fusion—typically resulting from the concatenation of data matrices from different modalities, before predictions are made on the subsequent matrix.

Mid-level/Feature fusion—individual data matrices undergo feature extraction; the resultant features are concatenated and used to make predictions [16,17].

Late/Decision fusion—individual machine learning models are trained on the data from each modality, and the decisions from each model are combined in some way, either through some form of averaging, or through the construction of second-layer (meta-learning) models, which are built upon the library of first-layer decision vectors, in a form similar to a “stacked generalisation” ensemble, which would typically exploit some degree of orthogonality in first level predictions, typically generated from a heterogeneous library of algorithm types and/or with varied data/feature engineering steps [18,19].

A variety of data fusion architectures have been explored in biosciences. Attempts to fuse data emerging from food sensor technologies may hamper the implementation of early- and mid-level architectures—differing data dimensionalities or scaling may limit the effectiveness of models trained on concatenated raw data or features without some intermediary scaling, as one data matrix may influence the model unfairly [17]. It was noted that while early- and mid-level data fusion are often recorded in the food analysis literature as producing better results than high-level approaches, a strength of the latter approach is the ease with which new modalities can be added to the overall model [20].

Smolinska et al. compare representative implementations of mid- and late fusion approaches for the prediction of gas composition, bitterness, and foam stability in beer using sensor data from gas and liquid chromatography coupled with mass spectrometry; their determination was that all approaches improved prediction accuracy over single-modality modelling and that late fusion performed consistently well in terms of accuracy, whilst the mid-level fusion approaches eased biological interpretation when fusing sensor data [17].

The aim of this research was to assess the comparative efficacy of individual high-throughput, minimally invasive sensor devices, and subsequent fusion of their outputs for the prediction of total viable counts of bacteria found in fresh poultry (chicken) and beef mince samples through the use of machine learning models trained on data recorded in aerobic and modified atmosphere or vacuum conditions. Data fusion workflows were implemented for combining the outputs of both sensor modalities (Fourier transform infrared and multispectral imaging), for the investigation of predictive performance versus single-sensor models.

## 2. Materials and Methods

### 2.1. Experimental Design, Sample Preparation, and Microbiological Analysis

#### 2.1.1. Chicken Thigh Samples

Fresh chicken thigh and minced beef samples were sourced from Greek meat distributors rapidly after preparation. Chicken thigh fillets were provided by the food company KOTINO, Athens, Greece. Sample storage and preparation are fully described by Fengou et al. [21].

Chicken samples were prepared and stored in either a vacuum-packaged or an aerobic state. The samples were stored at one of three isothermic temperatures for both packaging conditions, 0 °C, 5 °C (safe), or 10 °C (marginally abusive), and monitored at 24–48 h intervals, until visual deterioration of the samples became apparent.

Data for each storage condition were recorded in two batches, corresponding with meat sourcing on separate days, with a variable number of replicates (2–10) for each sampling condition in each batch.

Maximum storage time varied depending on the storage temperature and packaging condition: for the vacuum-packaged samples, those stored at 0 °C and 5 °C were monitored for a maximum of 240 h after collection, while those stored at 10 °C were monitored for a maximum of 120 h. For the aerobically packaged samples, those stored at 0 °C were monitored for a maximum of 216 h, those stored at 5 °C were monitored for a maximum of 192 h, and those at 10 °C were monitored for a maximum of 96 h.

#### 2.1.2. Beef Mince Samples

The beef mince samples were acquired specifically for the purpose of this study, sourced from a central butcher shop in Athens and transported under refrigeration to the laboratory within 30 min. Approximately 75–80 g portions were placed on Styrofoam trays and stored either in air or under modified atmosphere packaging (MAP) conditions (20% CO_2_/80% O_2_). For aerobic storage, the samples were covered with plastic food wrap typically used for domestic purposes. For MAP storage, the samples were sealed in plastic pouches with gas permeability rates of approximately 25 cm^3^/m^2^ per day for CO_2_ and 90 cm^3^/m^2^ per day for O_2_ at 20 °C and 50% relative humidity, using a Henkovac 1900 machine (Henkovac, ‘s-Hertogenbosch, Netherlands). The samples were stored at one of three isothermic temperatures for both packaging conditions (as above), and monitored at 24–48 h intervals, until visual deterioration of the samples became apparent. Data for each storage state were recorded in two batches, corresponding to meat sourcing on separate days, with a variable number of replicates (2–11) for each sampling condition in each batch.

Maximum storage time varied depending on the storage temperature and packaging condition: for the modified atmosphere meat samples, those stored at 0 °C and 5 °C were monitored with all sensor configurations for a maximum of 336 and 312 h after collection, respectively, and for 144 h for the samples stored at 10 °C. For the aerobically packaged samples, those stored at 0 °C were monitored for a maximum of 336 h, those stored at 5 °C were monitored for a maximum of 144 h, and those at 10 °C were monitored for a maximum of 120 h.

### 2.2. Microbiological Analysis

From each meat sample, 25 g portions were aseptically weighed into 400 mL sterile Stomacher bags (Seward Medical, London, UK) containing 225 mL of sterile quarter-strength Ringer’s solution (LabM Limited, Lancashire, UK) and homogenized for 60 s using a Lab Blender 400 (Seward Medical). Appropriate serial dilutions were then prepared using the same Ringer’s solution. From these dilutions, duplicate samples of 0.1 or 1 mL were either spread or mixed onto the following media: plate count agar (PCA, Biolife 4,021,452, Milan, Italy) for total viable counts (TVC), incubated at 30 °C for 48–72 h.

#### 2.2.1. Spectral Acquisition—Multispectral Imaging

MSI spectra for both the chicken and beef spoilage scenarios were captured using the VideometerLab platform (Videometer, Herlev, Denmark) [22], which captures information in a spread of 18 monochromatic wavelengths ranging from 405 nm to 970 nm, at intervals between 15 and 150 nm. The imaging data produced from the sensor are representative of two spatial dimensions and one wavelength dimension in a layered *width* × *height* × 18 data cube, with each layer representing the reflected response from illumination with each wavelength. To convert the data into a tabular form that can facilitate analysis with conventional data processing methods, a segmentation and averaging process was undertaken, wherein Canonical Discriminant Analysis (CDA) was performed, using the accompanying VideoMeter software version 2.12.39. In an averaging process described in Fengou et al. [23], the mean and standard deviation of intensity of reflectance across the target area was recorded. MSIF (multispectral imaging fluorescent) spectra are captured at 8 bands in the UV/blue light spectrum, ranging from 270 to 405 nm. Fluorescence bands were measured using a filter for combined reflectance/fluorescence.

#### 2.2.2. Spectral Acquisition—Fourier Transform Infrared Spectroscopy

FTIR spectra were recorded between 4000 and 400 cm^−1^ using a zinc selenide (ZnSe) HATR (Horizontal Attenuated Total Reflectance) crystal and an FTIR-6200 spectrometer (JASCO, Tokyo, Japan) equipped with a standard sample chamber, a triglycine sulphate (TGS) detector, and a Ge/KBr beamsplitter. The HATR crystal had a refractive index of 2.4 and a penetration depth of 2 μm at 1000 cm^−1^. Using the Spectra Manager™ Code of Federal Regulations (CFR) software version 2, spectra were collected by accumulating 100 scans with a resolution of 4 cm^−1^ and a total integration time of 2 min. A detailed description of the methodology employed in this work is presented by Argyri et al. [24].

### 2.3. Data Partitioning, Preprocessing, and Analysis

**Figure 1:**
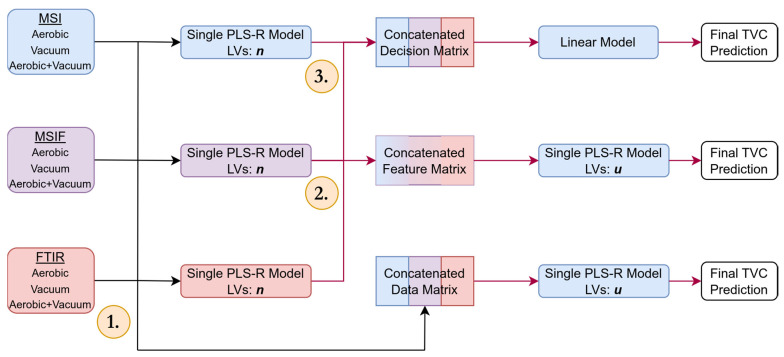
Graphical representation of early, feature (mid-) and decision (late) fusion approaches implemented using multispectral imaging (MSI), multispectral imaging fluorescent (MSIF), and Fourier transform infrared spectroscopy (FTIR) for total viable count (TVC) prediction. The upper examples represent decision fusion, wherein trained first-layer model predictions (3.) are passed to a linear model for combination of their predictions. The feature fusion approach uses first-layer partial least squares (PLS) models to generate projections for passing into a second-layer PLS model (2.), the number of latent variables ***u*** is tuned on these outputs. The lower approach represents early fusion, wherein the unprocessed data matrices are concatenated into a single matrix and standardised (1.) prior to passing to a subsequent PLS model. The number of latent variables retained was tuned in a manner similar to the feature fusion approach.

The overall aim of the data analysis segment of this research was to generate machine learning regression models for the prediction of TVC, using the sensor data collected under each experimental condition. For each meat type, single-sensor models were constructed, along with early, feature-, and decision-fusion approaches (Figure 1) for combining the outputs of the individual sensor devices with the intention of improving the overall predictive accuracy. Machine learning pipelines were constructed in the Python programming language (Version 3.11.7), using the Scikit-Learn framework (Version 1.3.0).

Two data partitioning methods were utilised for data analysis; the first was a batch-on-batch validation approach, in which models were trained and optimised with the first batch and tested with the second.

Pre-processing steps were undertaken on each training and testing set separately, to avoid data leakage. Observations were matched across datasets from different sensor modalities by using the observation naming scheme in the dataset. The resultant sample sizes for each storage condition are described in Table 1. For the MSI/MSIF datasets, Standard Normal Variate (SNV) filtering was undertaken to minimise baseline shifts in the dataset, similar to prior research [14,25]. For machine learning model construction, all mean and standard deviation information was used, as per prior research [23,26]. This resulted in a total of 36 features for the MSI data, and a total of 16 for MSIF. SNV was also applied to the chicken thigh FTIR data for both storage conditions, to remove baseline shifts in the dataset. Savinsky–Golay filtering was applied to FTIR data for both scenarios, with a first-order polynomial and a window size of 11 proving sufficient for smoothing without loss of useful information. A preliminary feature selection step was undertaken by restricting the FTIR spectral data to the wavelength range 2000–900 cm^−1^, as guided by the existing literature [14,27], and known as the ‘spoilage fingerprint region’ in many food group analyses, due to its correspondence to the excitation of polysaccharides, peptide bonds, and phosphate groups [28]. Within the processed FTIR data, a total of 1141 features were retained.

An alternative approach was implemented to provide the algorithms with larger calibration sets to assess the impact of the smaller training sample sizes used in the previous method, and to generalise the predictive out-of-sample performance achievable with the different strategies and thus offer greater statistical significance.

**Figure 2:**
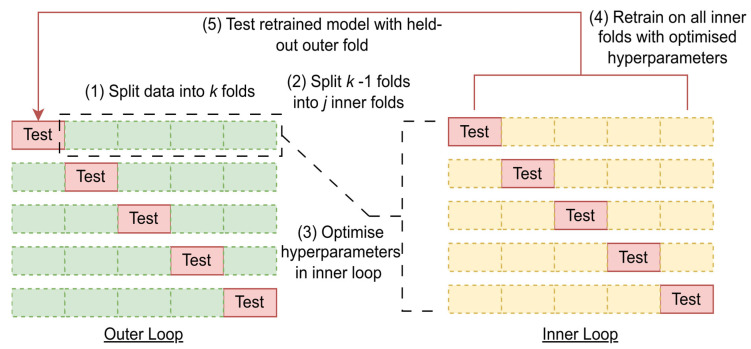
General schematic for a single fold of nested cross validation. This process is repeated for k folds. In the case of repetitions, the data are shuffled randomly before the same process occurs again for a new selection of k outer folds.

Ten repeats of five-fold nested cross-validation were undertaken with all model/fusion strategies under investigation, and all combinations of data—aerobic, vacuum/modified atmosphere, and combined conditions as before. This was intended to provide a more reliable estimate of model performance across the whole dataset. Each nested cross-validation round consisted of the random shuffling and splitting of the data into five folds, wherein five combinations of four folds were used for the nested hyper-parameter tuning process with an inner five-fold cross-validation procedure. The best candidate’s parameters in this nested validation were used to retrain on the whole inner dataset, and the model was tested with the respective held-out outer fifth fold. This whole process was repeated a further nine times, for a total of fifty assessments of performance, each made with random subsections of training and testing data. The utility of this process in minimising optimistic bias/overfitting in model assessment without split-to-split information leakage when compared to more conventional k-fold cross-validation has been acknowledged by Cawley and Talbot [29]. A schematic of this process is shown in Figure 2. Crucially, employment of the same random seed allows for all folds in the respective assessments using this technique to use the same series of random data splits. This allows for true comparison of model performance on a fold-to-fold basis, removing the opportunity for one algorithm to be assessed on an advantageous series of folds, while disadvantaging other algorithms with alternative splits. A similar approach has been employed by prediction of olive oil sensory descriptors, which coincidentally also explored the efficacy of early and feature-level fusion with PLS modelling [30].

#### 2.3.1. Machine Learning Methods

Partial least squares (PLS) methods were selected as the algorithm for the machine learning tasks. They are widely utilised in chemometric analysis due to their aptitude for dealing with the multicollinearity of data expected from spectroscopic sensor technologies, where the number of linearly independent columns of the resultant data matrix is far fewer than the total number of columns (low rank). Furthermore, the family of algorithms are often considered in situations where the number of predictor variables (independent variables) far exceed the number of observations, a situation known as the “small *n*, large *p*” problem, which can complicate some other types of machine learning methodologies that cannot account for sample matrices not possessing inverse or reciprocal forms [31]. Furthermore, previous research has shown that the algorithm is effective with similar meat spoilage scenarios [12,28,32]. A series of regression models were built, using the sensor-associated total viable counts (TVC) as the dependent variable.

For the batch-based performance assessment, the optimisation of machine learning models was conducted through the use of repeated k-fold cross-validation, in which the generalised performance of the selected machine learning model was assessed through 3 repetitions of dividing the dataset into 10 partitions; each fold was iteratively held out for evaluation, with remaining data used for model training. This methodology often provides more stable and robust performance estimates than Leave-One-Out Cross-Validation (LOOCV), which can yield higher variance due to the high correlation between its nearly identical training sets [33]. Optimisation of the PLS models took place in a grid search, wherein models built using varying numbers of latent variables (LVs) between 1 and 20 were assessed. Selection of optimal numbers of PLS LVs was based on minimisation of root mean square of error in cross-validation (RMSEcv). The exploration of the grid search space used for optimisation of hyper-parameters was performed through either randomized [34] or exhaustive search depending on the complexity of the model and search space; information on hyper-parameters is given in Table 2.

Individual PLS regression models were trained on each data type and storage condition, for a total of 6 single-storage-condition models for both the chicken and beef spoilage datasets. Additionally, single-sensor-modality models were trained on the data from both storage conditions, by combining the training partitions for each sensor type, resulting in 6 further models. Assessment of the complete models was achieved through use of the test set: root mean square of error in prediction (RMSEp) (log CFU/g) was recorded as the main performance metric for the test results, along with the coefficient of determination (R^2^) value. This metric is the proportion of variance in the dependent (target) variable explained by the model’s independent (predictor) variables [35]. A domain-specific metric was also recorded as an effort to categorise performance in otherwise regression-based analyses: “accuracy” is defined as the proportion of TVC predictions that fall within ±1 log CFU/g of observed values. This threshold of acceptability is based on prior microbiological growth research [14,15,23]. To explore the robustness of built models when encountering data from unseen conditions, the test sets from both storage conditions were used to assess model performance independently. Improvement of cross-condition performance was seen as a potential benefit for models incorporating data from both storage conditions.

#### 2.3.2. Multi-Sensor Fusion Strategies

Pipelines characterising the three main archetypes of data fusion (early, mid-/feature and late/decision) were developed to compare their microbial count predictive performance for both meat types and their respective storage conditions, in addition to the combination of conditions as previously described.

A naive early fusion approach for all conditions was examined, using the same procedure as described by Fengou et al. [23]. The heterogeneity of the data emerging from the project’s MSI, MSIF, and FTIR sensors necessitated preprocessing steps before combining their respective information. From each modality, the data were standardised (z-score normalised) before being concatenated into a single predictor matrix, then used as input for a single PLS regression model. To implement the mid-level approach, the previously developed single-sensor PLS models were used to transform the raw sensor data into the feature space of the respective models. These transformed features were horizontally concatenated into new matrices and used for the construction of a single new PLS model. As before, a repeated k-fold cross-validation scheme was used to determine the optimum number of latent variables. As described earlier, differences in dimensionality or scaling in early and mid-level fusion can lead to concatenated data matrices being dominated by the contributions of one modality [17]. A decision (late) fusion approach was developed alongside the former, which is, by nature, less prone to domination with information from a single modality.

The TVC prediction output from the single-sensor models for all experimental conditions (aerobic storage, vacuum storage, and combined) were concatenated into respective results matrices and served as predictor variables in a further regression layer used as a meta-learning step, with the observed TVC results serving as target variables, forming the final regression result. All three fusion methodologies are described in Figure 1.

The final approach employed the stacked generalisation methodology of training the meta-learner on the out-of-fold (OOF) predictions made by the first-layer models, described by Wolpert [18] and later by Laan et al. in their published strategy [36]. The training of the meta-learning layer on the OOF predictions of the first-layer models gives a representation of how the model may perform on unseen data. The meta-learner is thus trained on the predictions made by the first layer of the model, for all observations in the original training data, with the intention of reducing the risk of overfitting.

For this process, 20-fold cross-validation was used in the construction of the meta-learner training set, linear regression was used as the meta-learning algorithm, which was also used in Laan et al.’s simulation.

## 3. Results

The aim of study was to investigate the utility of integrating multi-sensor data, namely Fourier transform infrared spectroscopy (FTIR), multispectral imaging (MSI), and MSI fluorescence (MSIF), for the prediction of microbial spoilage in chicken and beef samples under various packaging and storage conditions. Over the following sections, the microbiological output, and output from the multi- and hyper-spectral sensors is outlined, as well as the predictive modelling performances obtained for each dataset using the microbiological count as output variables.

### 3.1. Microbial Assessment of Chicken Thigh Spoilage

**Figure 3:**
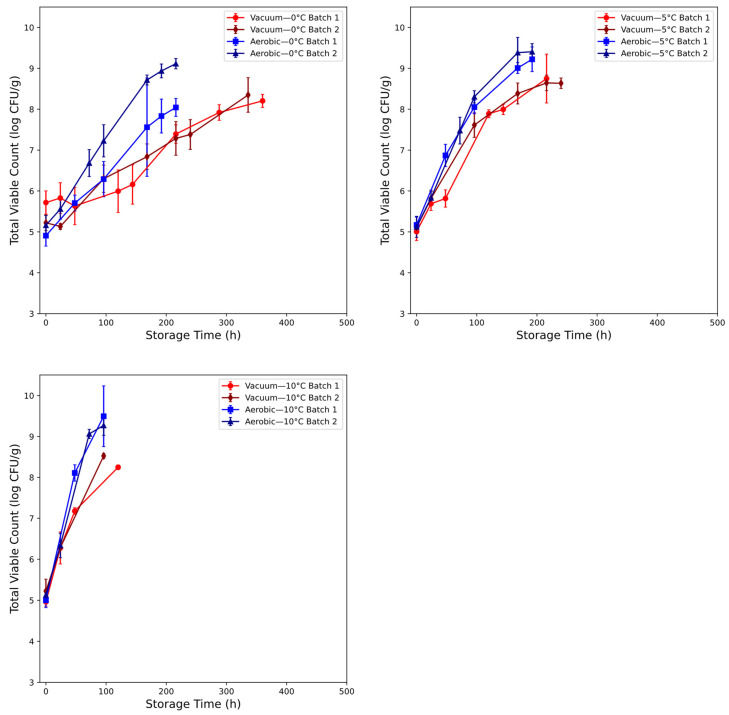
Mean and standard deviation of total viable count (TVC) recorded in chicken thigh for aerobic (batch 1: blue, batch 2: dark blue) and vacuum (batch 1: red, batch 2: dark red) packaging conditions at 0 °C, 5 °C, and 10 °C.

The microbial (TVC) populations for chicken spoilage are recorded in Figure 3. The initial TVC load in the sampled chicken thigh was shown to fall within the range of 4.5–6 log CFU/g. Storage temperature had an apparent large impact on the kinetics throughout storage. The initial disparity between batches for both atmospheric conditions appeared minimal, barring the mean initial measurements for batch 1 of the vacuum-packaged meat samples (5.72 versus 5.22 log CFU/g). Across all conditions, initial load appeared high—previous research conducted in similar conditions showed lower values of between 2 and 3.5 log CFU/g [14,28]. Storage in the safe conditions (0 °C and 5 °C) showed slowing of bacterial growth in vacuum-packaged meat when compared to aerobic storage—beyond 48 h at 5 °C and 72 h at 0 °C, batches of the former condition exhibited a widening TVC difference against the aerobically stored meat until the end of sampling. Mean TVC loads at 0 °C and 216 h were 7.39 and 7.29 log CFU/g for the vacuum condition and 8.04 and 9.11 for the aerobic condition. At 5 °C and 216 h, TVC was recorded at 8.75 and 8.63 for the vacuum condition. For the aerobic condition 5 °C and 168 h, TVC was recorded at 9.01 and 9.38. The marginally abusive condition (10 °C) exhibited the most severe microbial growth: aerobic counts at the end of sampling were 9.50 and 9.27 log CFU/g at 96 h, whereas the vacuum-packaged meat peaked at 8.25 and 8.53 log CFU/g at 120 h and 96 h for batch 1 and 2, respectively. Monitoring for all conditions was terminated at points past the 7–8 log CFU/g range previously reported as representative of spoilage in chicken [37].

### 3.2. Microbial Assessment of Beef Spoilage

**Figure 4:**
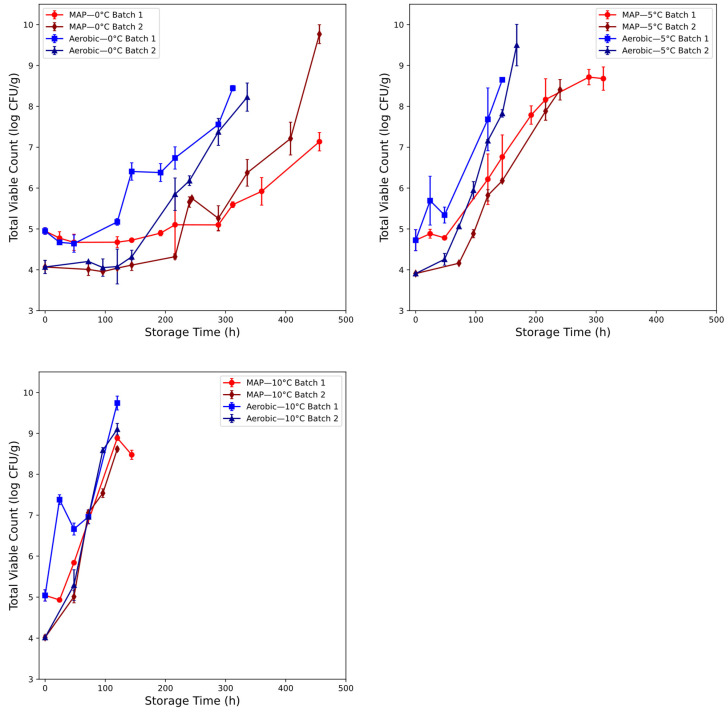
Mean and standard deviation of total viable count (TVC) recorded in beef mince for aerobic (batch 1: blue, batch 2: dark blue) and MAP (batch 1: red, batch 2: dark red) packaging conditions at 0 °C, 5 °C, and 10 °C.

The microbial (TVC) populations for beef mince spoilage are recorded in Figure 4. Initial TVC load for both packaging conditions show separation by batch, of between 0.5 and 1 log CFU/g. Batch 1 of the aerobic and modified atmosphere samples showed initial counts of 4.95, 4.72, and 5.04 log CFU/g at 0 °C, 5 °C, and 10 °C, respectively, whereas batch 2 showed counts of 4.07, 3.91, and 4.02 log CFU/g. This ~0.5–1 log CFU/g difference in initial TVC values seen in both aerobic and modified atmosphere packaging batches likely reflects the common origin of the batches and their simultaneous preparatory steps.

During the preparation of patties for storage, the mincing process is likely to distribute any surface contamination present in certain areas throughout the substrate and inoculate areas that were previously functionally sterile [38], in addition to decreasing the consistent baseline morphology of the underlying meat tissue, which may affect microbial growth in unpredictable ways. Despite the variability apparent in both the aerobic and modified atmospheric packaging (MAP) initial conditions, the aerobic batches tended towards a closer final count in both the 0 °C and 5 °C conditions. The MAP batches stored at 0 °C showed a reversal of highest counts at 216 h, possibly indicating a change in growth conditions, as batch 2 showed a declination of counts at 288 h, followed by a more severe, apparently exponential growth pattern onwards, counts in batch 2 of the MAP condition changed significantly from 7.21 log CFU/g at 408 h to 9.77 log CFU/g at 456 h.

### 3.3. Fourier Transform Infrared and Multispectral Imaging Measurements of Chicken Thigh and Beef Mince

**Figure 5:**
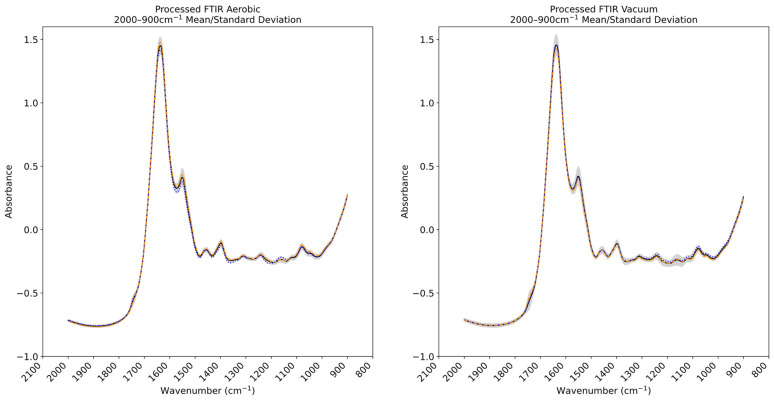
Plot showing mean spectral values of Fourier transform infrared spectroscopy (FTIR) data across the aerobically and vacuum-stored chicken thigh samples (solid black line). The standard deviation across the dataset is indicated by grey shading. Mean values are indicated for 0 °C/0 h (dotted blue line aerobic *n* = 10, vacuum *n* = 8) and 0 °C/216 h (dashed orange line aerobic *n* = 21, vacuum *n* = 8).

**Figure 6:**
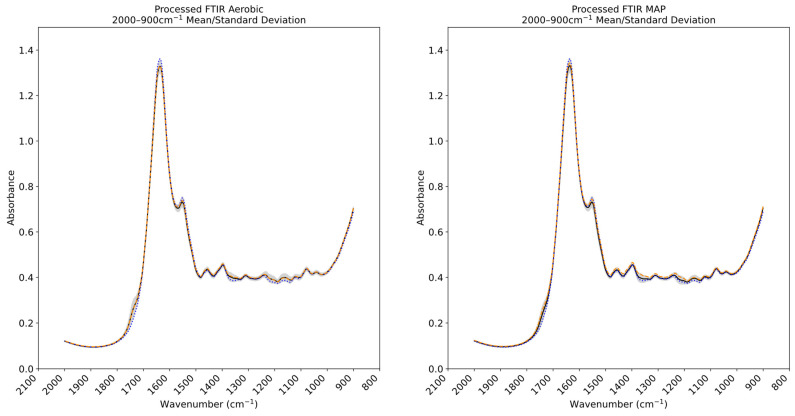
Plot showing mean spectral values of Fourier transform infrared spectroscopy (FTIR) data across the aerobically and modified atmosphere packaged (MAP) beef mince samples (solid black line). The standard deviation across the dataset is indicated by grey shading. Mean values are indicated for 0 °C/0 h (dotted blue line aerobic *n* = 4, MAP *n* = 4) and 0 °C/336 h (dashed orange line aerobic *n* = 4, MAP *n* = 4).

FTIR spectral plots corresponding to the mean and standard deviation recorded within the processed calibration sets of the chicken thigh and beef mince datasets are found in Figure 5 and Figure 6 Previous domain-specific research has highlighted the contribution of the 1800–1400 cm^−1^ band in spoilage analysis [14]. The spectra gathered through this research corroborate these contributions: large degrees of variance exist in the 1750–1400 cm^−1^ region indicated by the wide standard deviation margin in both aerobic and vacuum-stored samples, although this is partially obscured by the gradient of the peaks. The spectral regions peaking at ~1550 cm^−1^ and 1640 cm^−1^ have been associated with amide II vibrations [39], suggesting the presence of proteins and potential free amino acids, and is concordant with microbial spoilage-related proteolytic changes [40].

**Figure 7:**
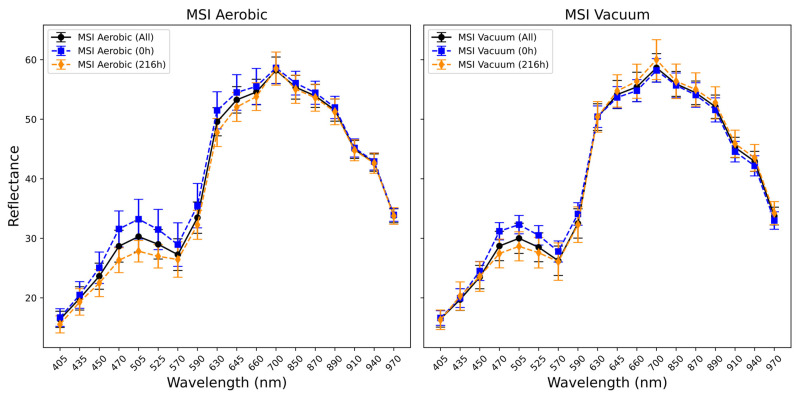
Plot showing mean and standard deviation of spectral values of Multispectral Imaging (MSI) data across the aerobically and vacuum-stored chicken thigh samples (solid black line). Mean and standard deviation values are also indicated for 0 °C/0 h (blue line aerobic *n* = 10, vacuum *n* = 8) and 0 °C/216 h (orange line aerobic *n* = 21, vacuum *n* = 8).

Figure 7 shows a large degree of variation in the aerobic packaged chicken thigh MSI 450–700 nm region for the 0 to 216 h window, and more limited variation in the 450–570 nm region for the vacuum-packaged chicken. Similar observations have been made for published chicken-based food products [15], with reflectance in this region attributed to pigments including myoglobin, oxymyoglobin, and metmyoglobin (570/590/630 nm) [41]. The more limited variation in the longer wavelength region may be indicative of the vacuum storage condition’s inhibitory effect on the metabolic processes of spoilage-causing microbiota and meat oxidation.

**Figure 8:**
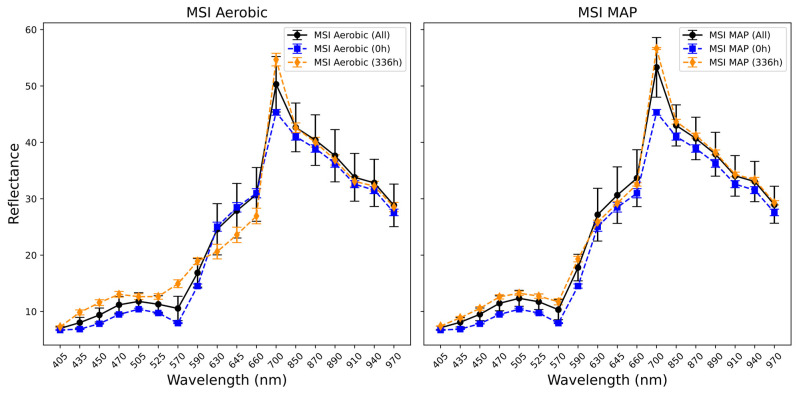
Plot showing mean and standard deviation of spectral values of MSI data across the aerobically and modified atmosphere-stored (MAP) minced beef samples (solid black line). Mean and standard deviation values are also indicated for 0 °C/0 h (blue line aerobic *n* = 4, MAP *n* = 4) and 0 °C/336 h (orange line aerobic *n* = 4, MAP *n* = 4).

Across the aerobic and modified atmosphere beef MSI measurements in Figure 8, the difference in reflectance between the 0 h and 336 h samples is more uniform across the wavelengths, except for a region in the aerobic data between 590 and 700 nm, in which the overall trend is inverted, with the 336 h samples showing a lesser degree of reflectance. This regional reduction in reflectance has been attributed to an effect similar to that observed with the chicken samples, namely colour changes (browning) due to the breakdown of meat pigment proteins [42].

### 3.4. Estimation of Microbial Activity in Chicken Thigh Using Machine Learning Analysis

**Figure 9:**
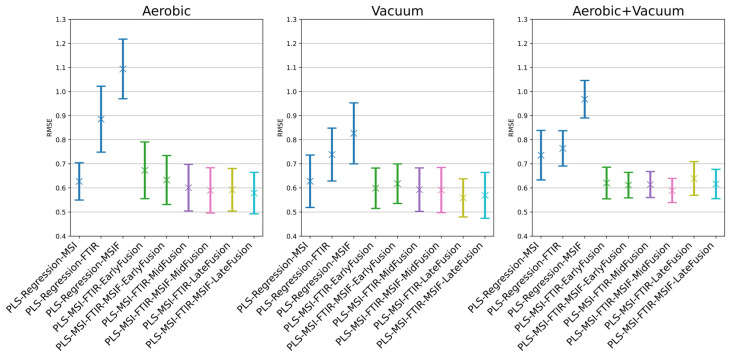
The root mean squared error of prediction of the repeated nested cross-validation process for chicken thigh data.

Performance metrics for the two data partitioning methodologies for chicken thigh samples are shown in Table 3 and Table 4, and Figure 9. Table 3 describes the performance of the complete model against the test set for the relevant modality and the “opposing” condition, for example, the recording of performance of a PLS model trained on MSI data from the aerobic packaging condition when tested with the test set from the vacuum-packaged meat. For the models trained with the concatenated training sets from both aerobic and vacuum conditions, performance was assessed with the respective test sets, giving performance metrics for each condition individually.

Performance of the complete models on the test partition that fell within the cross-validation standard deviation envelope was considered to be indicative of a better-generalised model. With the single sensor, single packaging condition models developed and validated in the batch-on-batch fashion, only the MSI models were found to perform within this envelope and achieve sub-1 log CFU/g performance; RMSEp values of 0.615 log CFU/g for the aerobic condition and 0.793 log CFU/g for the vacuum condition were recorded. These results can be seen in Supplementary Figure S1. For the MSI model built with the combined aerobic and vacuum training data, performance was found to be somewhat of a compromise between the predictive accuracies of the single-condition models when tested with the appropriate test set as seen in Supplementary Figure S5.

Models built with either the MSIF or FTIR data underperformed, with all single-condition models failing to achieve R^2^ ≥ 0.5. The two-condition FTIR and MSIF models narrowly reached this threshold, with FTIR achieving RMSEp performance of 1.131 log CFU/g for the aerobic condition and 1.030 log CFU/g for the vacuum condition. The two-modality (FTIR and MSI) late (decision) and mid-level (feature) approaches for all three conditions appeared to offer a greater degree of overall predictive utility than the single-modality models and those additionally incorporating the MSIF data. Improved test results were observed when comparing with the best-performing MSI models, with the single-condition MSI-FTIR aerobic model test set validation RMSE decreasing from 0.615 to 0.547 log CFU/g, and the corresponding vacuum model RMSE decreasing from 0.793 to 0.697 log CFU/g. These results are shown in Supplementary Figure S2.

The two-condition MSI-FTIR feature fusion models offered improvements in performance over the respective MSI model, with RMSE values improving from 0.731/0.809 log CFU/g for aerobic and vacuum data, respectively, to 0.678/0.752 log CFU/g and accuracy improving from 81.8%/72.2% to 89.4%/87.5%. Overall, addition of the MSIF data did not offer sizable performance benefits for the early and mid-/feature fusion models. Potential explanations could derive from the similarity in observational space captured by both the MSI and MSIF modalities, as both emerge from different configurations of the same sensor device.

Low-level fusion batch-on-batch validation results were unremarkable and were not suggestive of an alternative to the former approaches. As described in the prior literature [17,23], attempts to utilise sensor modalities with greatly differing dimensionalities may result in the larger “dominating” the useful effects of the smaller in resultant predictions. The naïve approach described here of simply concatenating the scaled dataset matrices does not offer any counter to the occurrence of these effects.

Previous food spoilage research has highlighted the importance of informative datasets that describe the plurality of food states in experimental scenarios when constructing robust models that generalise well to new data [14,32]. In addition to adopting a batch-derived partitioning approach, as described here, for the quantification of farmed sea bream microbial activity, Fengou et al. devised a random sampling approach for partitioning data into training and testing sets [32]. A more robust implementation of this approach is used here to generalise performance of the algorithms for out-of-sample data. Through the use of repeated cross-validation for 50 independent 80%/20% splits, average performance of the single-modality and fusion architectures is explored, in essence automating the training and testing process examined in the referenced work. This gives an indication of the relative differences in performance between models in mean RMSE, Accuracy, and R^2^, but also an indication of the generalisation and stability of performance across folds through the recording of standard deviation of these metrics. With limited training and testing data, evidence of model stability moving across folds is considered an important characteristic in food monitoring applications. Results for this process are shown graphically in Figure 9 and tabularly in Table 4. Across all conditions, it was found that on average, MSI outperformed the other single-modality models. The high mean and small standard deviation in the accuracy score for the MSI aerobic condition model suggests consistent placement of predictions in the ±1 log CFU/g window, regardless of RMSE performance, which the other two modalities are not able to achieve. The appropriate feature (mid-) and decision (late) fusion approaches were able to marginally exceed mean performance for aerobic MSI. For the vacuum condition models, only late fusion was able to significantly outperform the single-sensor modalities through increased performance across all three recorded metrics, while reducing the standard deviation recorded in each. The most statistically significant results were obtained with the combined aerobic and vacuum data due to the larger statistical sample: MSI/FTIR/MSIF feature (mid-) fusion was shown to offer the greatest degree of performance improvement over the single-sensor approaches in all metrics, showing an RMSEcv decrease of ~0.15 log CFU/g and a >7% increase in prediction accuracy over the best-performing single-sensor model (MSI), while substantially reducing standard deviation in both metrics, a result suggestive of a model with more predictive stability when encountering mixed condition data.

**Figure 10:**
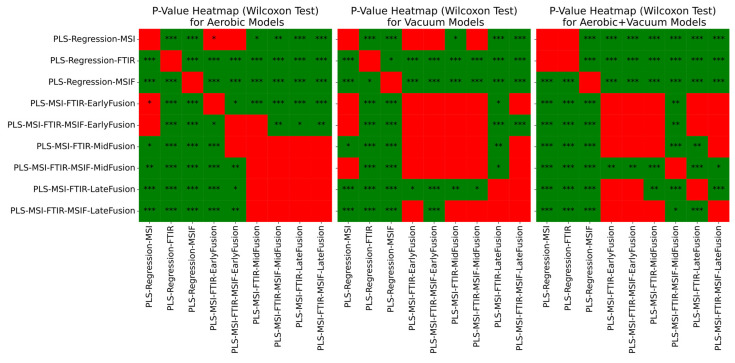
Wilcoxon signed-rank test significance levels for all repeated nested cross-validation scenarios for chicken thigh data, where ‘*’ = *p* < 0.05, ‘**’ = *p* < 0.01, and ‘***’ = *p* < 0.001. Results are coloured red where differences in median performance are insignificant (*p* > 0.05) or were significant in favour of a non-fusion approach.

The statistical significance of pairwise model comparisons was assessed using the Wilcoxon signed-rank test on RMSE values across cross-validation folds. To control for multiple comparisons, *p*-values were adjusted using the Holm–Bonferroni procedure [43], which maintains strong control of the family-wise error rate while providing greater statistical power than the standard Bonferroni correction. Significant increases in median RMSE performance across all scenarios were suggested when employing a late fusion approach, with Wilcoxon signed-rank test *p*-values < 0.001 (Figure 10). In the combined aerobic/vacuum scenario, all three fusion strategies displayed a significant improvement in median RMSE performance (*p*-values < 0.001). Interestingly, no significant difference in median performance was found across certain mid- and late fusion configurations in the aerobic and vacuum conditions (*p*-value > 0.05).

Whilst not entirely representative of the experimental conditions encountered in this research, Spyrelli et al.’s examination of the performance of PLS regression models using both FTIR and MSI for the prediction of spoilage in chicken achieved similar levels of performance—their conclusions were that the efficacy of their models were impacted by the inter-batch variability observed due to separation of meat sampling by four months [14]. Regardless, in their case, FTIR proved the most appropriate sensor type for informative model development. The poorer efficacy of FTIR models built in this research may be attributable to minimal preprocessing of the spectra, beyond restriction of the datasets to the 2000–900 cm^−1^ region as described, leading to models fitting on noisy data with a potential degree of confounding information.

### 3.5. Estimation of Microbial Activity in Beef Mince Using Machine Learning Analysis

**Figure 11:**
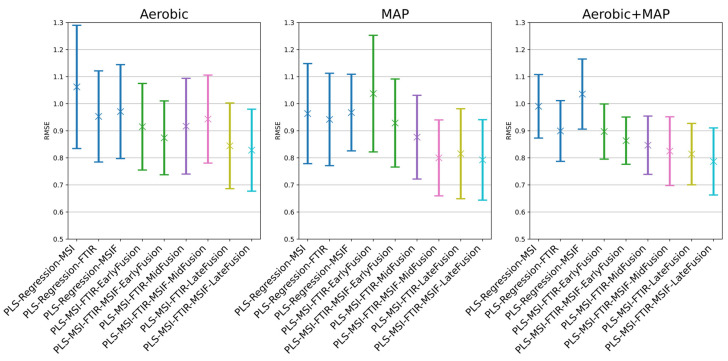
The root mean squared error of prediction of the repeated nested cross-validation process for beef mince data.

Performance metrics for beef mince data are shown in Table 5 for the batch-on-batch approach, and Figure 11/Table 6 for the alternative approach. Results for the batch-on-batch validation scheme appeared affected by the inter-batch variation visible in Figure 4. This is suggested by the strong intra-batch cross-validation performance exhibited by most single-sensor models. Batch-on-batch cross-validation RMSE performance across the decision fusion approaches is varied: in both the aerobic and combined conditions, the associated two- and three-sensor decision fusion models exceeded the cross-validation performance of all single-sensor models.

Predictions for the aerobic condition were improved with the MSI/FTIR late fusion model, with RMSE values decreasing from 1.306 to 1.167 log CFU/g and an accuracy improving from 45.7% to 56.5%. This aerobic performance was mirrored in the feature (mid-) fusion approach. The poor test set validation of the models is likely attributable to a combination of small training sets, along with the variation between the batches. None of the explored approaches were able to achieve RMSEp results lower than 1 log CFU/g. Only the combined aerobic and MAP MSIF model shows a performance approaching this threshold, with an RMSEp of 1.08 log CFU/g, however, the configuration appears unsuitable for prediction of the aerobic test TVC.

The results of this data partitioning approach highlight the high degree of variability between the batches used for training and testing the respective model types, stressing the importance of supplementation of the training data with further batches to construct more representative models that are applicable to “production” use cases. Supplementary Figure S3 highlights the bias present in the batch-on-batch single-sensor models. The models developed for the aerobic sensor modalities suggest a tendency of overestimation of bacterial counts, whereas the MSI and FTIR models developed for the modified atmosphere packaging condition show poor generalisation to the test batch.

**Figure 12:**
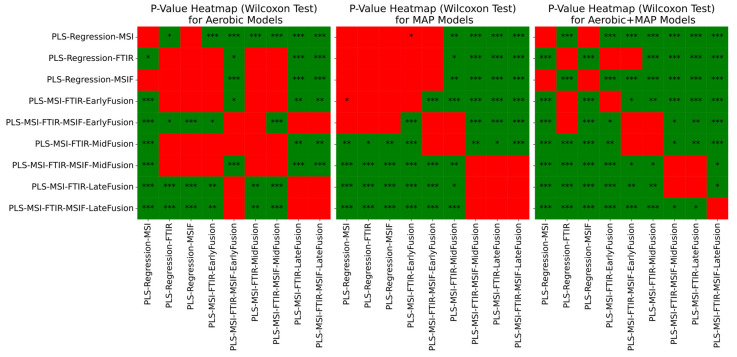
Wilcoxon signed-rank test significance levels for all repeated nested cross-validation scenarios for beef mince data, where ‘*’ = *p* < 0.05, ‘**’ = *p* < 0.01, and ‘***’ = *p* < 0.001. Results are coloured red where differences in median performance are insignificant (*p* > 0.05) or were significant in favour of a non-fusion approach.

In a similar manner to the chicken spoilage scenario, repeated nested cross-validation was used to assess the generalised performance of the three tiers of fusion approach when compared to the corresponding single-sensor models, albeit with a lesser degree of confidence due to the wider standard deviation envelope. A general downward trend was visible in Figure 11 in mean and standard deviation of RMSE performance when moving from single sensors towards decision (late) fusion methods. Wilcoxon signed-rank tests were used to aid the quantification of the significance of differences in median performance with minimal assumptions as to the underlying distribution of results (Figure 12), with Holm–Bonferroni correction applied. For the aerobic and combined aerobic and MAP conditions, FTIR performance was significantly different to MSI, with Wilcoxon signed-rank *p*-value < 0.05. Across all scenarios, MSI/FTIR(/MSIF) decision fusion achieved a significant improvement in performance against single-sensor approaches, with *p*-values of at most 0.001. The performance metrics for the combined aerobic and MAP condition (Table 6) suggest the most convincing improvements to predictive performance by adopting a sensor fusion approach: MSI/FTIR/MSIF mid-/late fusion show an increase in mean accuracy of between 6 and 8% over FTIR alone.

## 4. Discussion and Conclusions

The results of this comparative performance assessment of different data fusion approaches in two meat spoilage scenarios suggest in a majority of cases enhanced predictive capability and generalisation to new data when compared to models built with individual constituent sensor device data. At the time of writing, fusion of multispectral imaging and Fourier transform infrared in the context of meat spoilage assessment has not seen significant publication.

Results from the batch validation feature fusion and decision fusion pipelines showed that the latter’s results are in most cases on-par with feature fusion, and in the case of chicken spoilage, superior performance is observed. The performance of the fusion approaches was corroborated in the repeated nested cross-validation results: a general trend is an improvement in mean RMSE performance when compared to the single-sensor approaches, alongside a reduction in standard deviation, indicating more stable performance in the recorded fold results. For the individual sensor approaches explored for chicken spoilage analysis, MSI performance was in most cases superior to FTIR and MSI with fluorescence (MSIF), and in some cases performed similarly to the equivalent fusion approaches assessed. For the beef spoilage scenario, the individual sensor results were more unstable, with FTIR narrowly achieving better RMSE performance, with decision fusion methods indicated as offering the most significant performance improvement. It is anticipated that refinement of these feature and late fusion methods, through more consideration of the fusion strategy, or, in the case of the latter, additions to the first-layer (expert) model library or reconfiguration of the meta-learning layer will result in improved performance, bringing the strategy more in line with stacked generalisation approaches. Modification of the hyper-parameter tuning regime with alternative approaches, such as Bayesian methods, may accelerate convergence on optimal configurations.

Whilst widely explored in a variety of contexts outside of food analysis, sensor/data fusion has seen limited utilisation in the area of meat spoilage quantification. Previous attempts have been made in the analysis of minced meat spoilage in Fengou et al. [23], which used naïve early fusion as previously described, the results of which suggested further research and optimisation was a potential avenue to explore for improvement of spoilage prediction. Additional data fusion efforts have been made in the prediction of other meat quality metrics, such as moisture content in freeze-thawed pork [44], in which adoption of decision fusion approaches similar to that implemented here achieved a notable enhancement of predictive utility over the models using single-sensor modalities in isolation. The exploration of fusion methodologies in the context of food spoilage is timely, as increasing affordability of many high-throughput, minimally invasive sensor types is likely to prompt increased adoption throughout production and distribution pipelines. While unlikely to offer an accuracy matching established lab testing protocol in isolation, they offer clear benefits in terms of responsiveness, portability, and, economic optimisation through minimisation of food wastage. Fusion of these sensor outputs is one possible avenue with which to improve upon predictive accuracy and reliability; interpretation of this growing wealth of multi-modal food monitoring data will likely lead to more informative, robust models for a variety of food control tasks.

The machine learning models generated in this work have corroborated existing research in the utilisation of sensor technologies for microbial count prediction, whilst also highlighting the potential performance improvements achievable by implementing a data fusion approach for combining their predictive power. The performance of these approaches serves as a suggestion of directions for the optimisation of high-throughput sensor performance in meat analysis, as model training has been constrained with small sample sizes. Even so, the comparison of multiple architectures on an “even footing” suggest performance advantages obtainable by adopting fusion approaches in this domain.

Regarding the efficacy of individual sensors applied in this research, it is likely that further work in the data preparatory stage may positively impact the predictive power of resultant models. Comparison with existing research shows similar data processing procedures were used for MSI and FTIR spectral preparation [14,15,28], namely the application of SNV and filtering techniques for preprocessing spectral datasets. However, some sources demonstrate optimisation and feature selection techniques for highly dimensional data, such as that from FTIR, that may improve results even for machine learning algorithms that are capable of handling some degree of collinearity, such as partial least squares techniques (PLS). Use of these methods must account for the data fusion context; some methods, such as recursive feature elimination and genetic algorithm- (GA) derived techniques have shown promise in optimising the number of features available to subsequent algorithms [45]. However, these methods result in the removal of information that is not informative in a solitary context, but may become informative with the addition of additional information provided by other sensor modalities [17].

In the interest of improving confidence in decisions increasingly informed by the use of high-throughput sensor technologies in the food analysis sector, more well-informed machine learning predictions may be made by incorporating the predictive power from multiple sensor modalities, reducing uncertainty by forming a more complete view of biological state though the union of each sensor’s observational spaces. In practice, these sensor observations could be made throughout the food production and distribution pipeline, with a combination of devices installed in a permanent fashion with specialist oversight, and those deployed portably at the food business operator level [4]. The successful summation and fusion of this food state information in a real-world situation is dependent on the success of analysts in accounting for the information collected across differences in sampling times and storage and distribution conditions. As such, appropriate decision support pipelines may be necessary to handle the influx of information and facilitate predictive model construction and inference in a semi-automated fashion.

Further work is required to refine fusion techniques suitable for the food analysis domain, through the exploration of different combinations of machine learning algorithms and methodologies, with larger quantities of experimental data with which to build more dependable models. In conclusion, the approaches demonstrated here may serve as an indicator of potential utility of all three general types of sensor fusion in real-world food analysis scenarios.

## Figures and Tables

**Table 1:** Table of calibration/cross-validation (CV) and test set sizes for chicken and beef spoilage scenarios using batch validation approach. In addition to the aerobic and vacuum/MAP conditions, a further calibration/test split was synthesised for each meat type, by independently combining the calibration and test sets of the aerobic/vacuum-packaged chicken and aerobic/modified atmosphere packaged beef, to assess the performance of models constructed with data from both respective packaging conditions.

Partition			
**Chicken Spoilage**	**Aerobic condition**	**Vacuum condition**	**Combined conditions**
Calibration	70	65	135
CV	70	65	135
Prediction	66	72	138
Repeated Nested CV	136	137	273
**Beef Spoilage**	**Aerobic condition**	**MAP condition**	**Combined conditions**
Calibration	46	57	103
CV	46	57	103
Prediction	46	56	102
Repeated Nested CV	92	113	205

**Table 2:** Grid search space for the repeated nested cross-validation approach. In the case of random search for MSI-MSI mid-fusion and MSI-MSIF-FTIR mid-/late fusion, 1500 (~37% coverage), 2500 (~12% coverage) and 400 (~30% coverage) iterations were performed per inner grid search, respectively, with each parameter sampled from a uniform distribution. All other model types used exhaustive grid search. MSI: multi spectral imaging. MSIF: multispectral imaging fluorescent. FTIR: Fourier transform infrared spectroscopy (FTIR).

Model	Range
*Single-Sensor Models and Mid-/Late Fusion Base Models*	
MSI—pls__n_components	2–17
FTIR—pls__n_components	2–20
MSIF—pls__n_components	2–7
*Early Fusion Models*	
pls__n_components	2–20
*Mid Fusion Models*	
pls__n_components	2–17

**Table 3:** Performance metrics for PLS-R models built for the purpose of estimation of bacterial total viable count (log CFU/g TVC) in chicken thigh samples using batch-on-batch validation (aerobic *n* = 70, vacuum *n* = 65, combined *n* = 135). MSI: multi spectral imaging. MSIF: multispectral imaging fluorescent. FTIR: Fourier transform infrared spectroscopy (FTIR).

Model		LOOCV Performance		Aerobic Performance			Vacuum Performance		
		RMSEcv (SD)	LVs (Meta LVs)	RMSEp	Acc %	R^2^	RMSEp	Acc %	R^2^
MSI	Aerobic	0.6779 (0.1664)	9	0.6147	86.3636	0.8604	1.4145	56.9444	−0.1725
	Vacuum	0.5199 (0.1702)	4	0.8956	75.7576	0.7037	0.7933	75	0.6312
	A + V	0.6928 (0.1311)	6	0.7311	81.8182	0.8025	0.809	72.2222	0.6165
MSIF	Aerobic	0.8329 (0.2677)	3	2.2473	30.303	−0.8655	1.7561	41.6667	−0.8073
	Vacuum	0.7304 (0.3058)	5	1.6805	42.4242	−0.0432	1.0976	63.8889	0.2941
	A + V	0.8333 (0.1779)	6	1.5469	42.4242	0.1161	0.9222	70.8333	0.5016
FTIR	Aerobic	0.8936 (0.2631)	6	1.604	53.0303	0.0496	1.7679	50	−0.8315
	Vacuum	0.6321 (0.1211)	6	1.4657	56.0606	0.2064	1.2924	62.5	0.0212
	A + V	0.8764 (0.1789)	7	1.131	63.6364	0.5275	1.0304	68.0556	0.3779
MSI +FTIR	*Late (Decision) Fusion*								
	Aerobic	0.6421 (0.2086)	9,6	0.5466	93.9394	0.8896	1.3863	52.7778	−0.1263
	Vacuum	0.4829 (0.1616)	4,6	0.9821	63.6364	0.6437	0.6971	81.9444	0.7152
	A + V	0.6314 (0.1126)	6,7	0.6779	84.8485	0.8302	0.6959	84.7222	0.7162
	*Mid- (Feature) Fusion*								
	Aerobic	0.6483 (0.1857)	9,6 (6)	0.5607	93.9394	0.8838	1.3778	56.9444	−0.1124
	Vacuum	0.4753 (0.1293)	4,7 (5)	0.9415	66.6667	0.6725	0.6638	84.7222	0.7418
	A + V	0.6049 (0.1400)	6,7 (6)	0.6782	89.3939	0.8301	0.752	87.5	0.6686
	*Early Fusion*								
	Aerobic	0.8334 (0.2347)	6	1.2906	72.7273	0.3847	1.7791	44.4444	−0.8548
	Vacuum	0.5429 (0.1905)	7	1.3672	53.0303	0.3094	1.2745	69.4444	0.0481
	A + V	0.7672 (0.1736)	6	1.1962	66.6667	0.4713	1.2714	58.3333	0.0527
MSI +FTIR +MSIF	*Late (Decision) Fusion*								
	Aerobic	0.6399 (0.1930)	9,6,3	0.6533	87.8788	0.8424	0.994	69.4444	0.421
	Vacuum	0.4997 (0.1603)	4,6,5	0.9978	63.6364	0.6323	0.6918	83.3333	0.7195
	A + V	0.5943 (0.1210)	6,7,6	0.7611	80.303	0.786	0.652	84.7222	0.7509
	*Mid- (Feature) Fusion*								
	Aerobic	0.6269 (0.1938)	9,6,3 (6)	0.5907	90.9091	0.8711	1.0228	65.2778	0.387
	Vacuum	0.5026 (0.1956)	4,6,5 (5)	1.0467	62.1212	0.5952	0.701	83.3333	0.712
	A + V	0.5748 (0.1300)	6,7,6 (5)	0.7818	75.7576	0.7742	0.7544	80.5556	0.6665
	*Early Fusion*								
	Aerobic	0.7731 (0.2164)	6	1.0802	72.7273	0.569	1.6575	44.4444	−0.6099
	Vacuum	0.5472 (0.2002)	7	1.3565	45.4545	0.3203	1.2171	70.8333	0.132
	A + V	0.6884 (0.1774)	6	1.1749	63.6364	0.4901	1.0544	63.8889	0.3486

**Table 4:** Generalised performance metrics for PLS-R models built for the purpose of estimation of bacterial total viable count (log CFU/g TVC) in chicken thigh samples using repeated nested cross-validation (10 × 5 folds).

Model	RMSE Mean	RMSE SD	Acc % Mean	Acc % SD	R^2^ Mean	R^2^ SD
Aerobic Models						
PLS-Regression-MSI	0.6270	0.0766	89.4921	5.5495	0.8313	0.0426
PLS-Regression-FTIR	0.8853	0.1355	75.4497	8.1551	0.6548	0.1428
PLS-Regression-MSIF	1.0940	0.1226	61.1323	8.1930	0.4876	0.1170
PLS-MSI-FTIR-EarlyFusion	0.6731	0.1165	86.3942	6.6007	0.8013	0.0757
PLS-MSI-FTIR-MSIF-EarlyFusion	0.6328	0.1007	88.5344	6.4148	0.8251	0.0590
PLS-MSI-FTIR-MidFusion	0.6010	0.0956	90.4233	6.0550	0.8432	0.0538
PLS-MSI-FTIR-MSIF-MidFusion	0.5899	0.0930	90.0026	5.7044	0.8494	0.0504
PLS-MSI-FTIR-LateFusion	0.5923	0.0875	91.6270	5.3435	0.8485	0.0479
PLS-MSI-FTIR-MSIF-LateFusion	0.5785	0.0851	91.7619	4.7053	0.8554	0.0471
Vacuum Models						
PLS-Regression-MSI	0.6276	0.1076	89.1217	6.4258	0.7432	0.1014
PLS-Regression-FTIR	0.7385	0.1087	84.9841	5.0404	0.6475	0.1137
PLS-Regression-MSIF	0.8266	0.1254	78.0291	6.6680	0.5587	0.1415
PLS-MSI-FTIR-EarlyFusion	0.5988	0.0829	90.3651	5.3492	0.7688	0.0691
PLS-MSI-FTIR-MSIF-EarlyFusion	0.6176	0.0813	89.3439	5.0660	0.7537	0.0755
PLS-MSI-FTIR-MidFusion	0.5928	0.0893	91.1164	5.4086	0.7717	0.0801
PLS-MSI-FTIR-MSIF-MidFusion	0.5912	0.0927	90.1746	6.0481	0.7727	0.0804
PLS-MSI-FTIR-LateFusion	0.5586	0.0782	91.3201	5.5276	0.7977	0.0698
PLS-MSI-FTIR-MSIF-LateFusion	0.5691	0.0941	91.0291	5.5210	0.7891	0.0802
Aerobic + Vacuum Models						
PLS-Regression-MSI	0.7358	0.1018	84.2222	4.4987	0.7344	0.1007
PLS-Regression-FTIR	0.7641	0.0727	82.1293	4.5691	0.7218	0.0599
PLS-Regression-MSIF	0.9682	0.0771	68.9360	6.2140	0.5536	0.0817
PLS-MSI-FTIR-EarlyFusion	0.6203	0.0650	89.7461	4.2534	0.8159	0.0431
PLS-MSI-FTIR-MSIF-EarlyFusion	0.6116	0.0524	90.0094	3.5852	0.8221	0.0324
PLS-MSI-FTIR-MidFusion	0.6141	0.0534	90.4431	3.2687	0.8198	0.0393
PLS-MSI-FTIR-MSIF-MidFusion	0.5892	0.0494	91.5091	3.2946	0.8346	0.0323
PLS-MSI-FTIR-LateFusion	0.6392	0.0693	88.9044	3.3687	0.8029	0.0561
PLS-MSI-FTIR-MSIF-LateFusion	0.6163	0.0603	89.8949	3.7594	0.8180	0.0440

**Table 5:** Performance metrics for PLS-R models built for the purpose of estimation of bacterial total viable count (log CFU/g TVC) in beef mince samples using batch-on-batch validation (aerobic *n* = 46, vacuum *n* = 57, combined *n* = 103). MSI: multispectral Imaging. MSIF: multispectral imaging fluorescent. FTIR: Fourier transform infrared spectroscopy (FTIR).

Model		LOOCV Performance		Aerobic Performance			MAP Performance		
		RMSEcv (SD)	LVs (Meta LVs)	RMSEp	Acc %	R^2^	RMSEp	Acc %	R^2^
MSI	Aerobic	0.7170 (0.2614)	4	1.3048	45.6522	0.4326	1.8746	37.5000	−0.1164
	MAP	0.5578 (0.1258)	5	1.3074	56.5217	0.4303	1.4027	58.9286	0.3749
	A + M	0.7583 (0.1668)	5	1.4338	47.8261	0.3148	1.3526	55.3571	0.4188
MSIF	Aerobic	0.9630 (0.2673)	5	1.7839	30.4348	−0.0614	1.2002	51.7857	0.5422
	MAP	0.8023 (0.2010)	3	2.5896	19.5652	−1.2368	1.3251	42.8571	0.4419
	A + M	0.8441 (0.2095)	7	1.8263	30.4348	−0.1124	1.0808	62.5000	0.6288
FTIR	Aerobic	0.7764 (0.2573)	9	1.3851	52.1739	0.3606	2.4253	39.2857	−0.8688
	MAP	0.8309 (0.3005)	15	1.4396	47.8261	0.3093	1.6286	37.5000	0.1574
	A + M	0.7666 (0.1561)	16	1.5534	50.0000	0.1958	1.7130	44.6429	0.0678
MSI +FTIR	*Late (Decision) Fusion*								
	Aerobic	0.5854 (0.2300)	4,9	1.1663	56.5217	0.5467	2.0272	44.6429	−0.3056
	MAP	0.5502 (0.1436)	5,15	1.2636	63.0435	0.4678	1.2846	62.5000	0.4757
	A + M	0.6220 (0.1271)	5,16	1.2273	56.5217	0.4980	1.4416	53.5714	0.3398
	*Mid- (Feature) Fusion*								
	Aerobic	0.5893 (0.2259)	4,9 (3)	1.1896	58.6957	0.5284	2.0653	41.0714	−0.3551
	MAP	0.5074 (0.1135)	5,15 (5)	1.2361	67.3913	0.4907	1.2968	66.0714	0.4657
	A + M	0.5145 (0.0929)	5,16 (10)	1.4185	54.3478	0.3290	1.6726	44.6420	0.1110
	*Early Fusion*								
	Aerobic	0.6197 (0.2245)	5	1.8869	41.3043	−0.1866	1.7650	25.0000	0.0103
	MAP	0.5767 (0.1555)	7	2.4270	17.3913	−0.9631	1.9378	30.3571	−0.1930
	A + M	0.6814 (0.1456)	6	1.4526	45.6522	0.2968	1.3907	39.2857	0.3856
MSI +FTIR +MSIF	*Late (Decision) Fusion*								
	Aerobic	0.5907 (0.2308)	4,9,5	1.1186	58.6957	0.5827	1.8653	51.7857	−0.1058
	MAP	0.5535 (0.1447)	5,15,3	1.2515	56.5217	0.4776	1.2531	62.5000	0.5010
	A + M	0.6282 (0.1277)	5,16,7	1.1865	58.6957	0.5304	1.4244	53.5714	0.3551
	*Mid- (Feature) Fusion*								
	Aerobic	0.5793 (0.1983)	4,9,5 (3)	1.1031	60.8696	0.5945	1.7455	51.7857	0.0321
	MAP	0.5457 (0.1266)	5,15,3 (5)	1.3075	60.8696	0.4302	1.3970	66.0714	0.3800
	A + M	0.6650 (0.1410)	5,16,7 (4)	1.2013	56.5217	0.5186	1.4952	55.3571	0.2894
	*Early Fusion*								
	Aerobic	0.6411 (0.2394)	5	1.7345	43.4783	−0.0034	1.6092	26.7857	0.1770
	MAP	0.6035 (0.1595)	7	2.1515	21.7391	−0.5440	1.6677	35.7143	0.1161
	A + M	0.6963 (0.1450)	6	1.3967	52.1739	0.3494	1.3136	44.6429	0.4516

**Table 6:** Generalised performance metrics for PLS-R models built for the purpose of estimation of bacterial total viable count (log CFU/g TVC) in beef mince samples using repeated nested cross-validation (50 folds). MSI: multispectral imaging. MSIF: multispectral imaging fluorescent. FTIR: Fourier transform infrared spectroscopy (FTIR).

Model	RMSE Mean	RMSE SD	Acc % Mean	Acc % SD	R^2^ Mean	R^2^ SD
Aerobic Models						
PLS-Regression-MSI	1.0622	0.2255	69.4444	10.2883	0.5332	0.1809
PLS-Regression-FTIR	0.9530	0.1668	73.0526	10.3674	0.6258	0.1233
PLS-Regression-MSIF	0.9709	0.1719	70.2515	8.6537	0.6106	0.1326
PLS-MSI-FTIR-EarlyFusion	0.9149	0.1584	76.0117	9.7809	0.6539	0.1158
PLS-MSI-FTIR-MSIF-EarlyFusion	0.8739	0.1351	78.7368	6.6358	0.6859	0.1021
PLS-MSI-FTIR-MidFusion	0.9170	0.1752	75.6842	9.5596	0.6491	0.1290
PLS-MSI-FTIR-MSIF-MidFusion	0.9432	0.1610	74.3333	7.7958	0.6309	0.1333
PLS-MSI-FTIR-LateFusion	0.8444	0.1567	78.9181	9.7589	0.7057	0.1024
PLS-MSI-FTIR-MSIF-LateFusion	0.8284	0.1499	76.9649	9.4674	0.7181	0.0908
MAP Models						
PLS-Regression-MSI	0.9635	0.1831	74.7352	9.1533	0.6241	0.1577
PLS-Regression-FTIR	0.9419	0.1690	73.2846	9.3656	0.6419	0.1374
PLS-Regression-MSIF	0.9674	0.1402	70.5336	10.7800	0.6275	0.1141
PLS-MSI-FTIR-EarlyFusion	1.0374	0.2133	73.1383	9.5623	0.5593	0.1954
PLS-MSI-FTIR-MSIF-EarlyFusion	0.9287	0.1611	76.6680	9.0988	0.6476	0.1481
PLS-MSI-FTIR-MidFusion	0.8764	0.1532	76.9091	8.2672	0.6938	0.1011
PLS-MSI-FTIR-MSIF-MidFusion	0.7999	0.1389	79.9684	8.1167	0.7413	0.0943
PLS-MSI-FTIR-LateFusion	0.8151	0.1646	82.7747	7.7723	0.7315	0.1094
PLS-MSI-FTIR-MSIF-LateFusion	0.7924	0.1471	81.9130	8.9743	0.7491	0.0838
Aerobic + MAP Models						
PLS-Regression-MSI	0.9904	0.1162	73.0244	7.0759	0.6210	0.0883
PLS-Regression-FTIR	0.8992	0.1111	75.7561	5.9825	0.6898	0.0650
PLS-Regression-MSIF	1.0356	0.1283	69.8537	7.1320	0.5817	0.1172
PLS-MSI-FTIR-EarlyFusion	0.8972	0.1010	77.5122	5.6106	0.6906	0.0643
PLS-MSI-FTIR-MSIF-EarlyFusion	0.8635	0.0865	77.6098	6.0670	0.7125	0.0600
PLS-MSI-FTIR-MidFusion	0.8467	0.1067	79.2683	5.8384	0.7234	0.0677
PLS-MSI-FTIR-MSIF-MidFusion	0.8247	0.1257	81.4634	6.6528	0.7369	0.0726
PLS-MSI-FTIR-LateFusion	0.8138	0.1123	81.9024	5.5638	0.7457	0.0597
PLS-MSI-FTIR-MSIF-LateFusion	0.7867	0.1227	83.8049	6.2233	0.7599	0.0704

## Data Availability

The original contributions presented in the study are included in the article/Supplementary Material, further inquiries can be directed to the corresponding author.

## Supplementary Materials

The following supporting information can be downloaded at: https://www.mdpi.com/article/10.3390/foods14091613/s1, Figure S1. Test performance plots for single-sensor, single-packaging-condition chicken thigh spoilage prediction models built using MSI, FTIR and MSIF datasets. MSI: Multi Spectral Imaging. MSIF: Multispectral Imaging Fluorescent. FTIR: Fourier Transform Infrared Spectroscopy (FTIR). Figure S2. Test performance plots for two/three-sensor, single-packaging-condition chicken thigh spoilage prediction models built using MSI, FTIR and MSIF datasets, built using the late (decision) fusion approach. MSI: Multi Spectral Imaging. MSIF: Multispectral Imaging Fluorescent. FTIR: Fourier Transform Infrared Spectroscopy (FTIR). Figure S3. Test performance plots for single-sensor, single-packaging-condition beef mince spoilage prediction models built using MSI, FTIR and MSIF datasets. MSI: Multi Spectral Imaging. MSIF: Multispectral Imaging Fluorescent. FTIR: Fourier Transform Infrared Spectroscopy (FTIR). Figure S4. Test performance plots for two/three-sensor, single-packaging-condition beef mince spoilage prediction models built using MSI, FTIR and MSIF datasets, built using the late (decision) fusion approach. MSI: Multi Spectral Imaging. MSIF: Multispectral Imaging Fluorescent. FTIR: Fourier Transform Infrared Spectroscopy (FTIR). Figure S5. Test performance plots for single-sensor, two-packaging-condition chicken thigh spoilage prediction models built using MSI, FTIR and MSIF datasets. Figure S6. Test performance plots for two/three-sensor, two-packaging-condition chicken thigh spoilage prediction models built using MSI, FTIR and MSIF datasets, built using the late (decision) fusion approach. MSI: Multi Spectral Imaging. MSIF: Multispectral Imaging Fluorescent. FTIR: Fourier Transform Infrared Spectroscopy (FTIR). Figure S7. Test performance plots for single-sensor, two-packaging-condition beef mince spoilage prediction models built using MSI, FTIR and MSIF datasets. Figure S8. Test performance plots for two/three-sensor, two-packaging-condition beef mince spoilage prediction models built using MSI, FTIR and MSIF datasets, built using the late (decision) fusion approach. MSI: Multi Spectral Imaging. MSIF: Multispectral Imaging Fluorescent. FTIR: Fourier Transform Infrared Spectroscopy (FTIR).

## Abbreviations

The following abbreviations are used in this manuscript:

CFU	Colony Forming Units
FTIR	Fourier Transform Infrared
MSI(F)	Multispectral Imaging (Fluorescent)
PLS	Partial Least Squares
RMSE	Root Mean Squared Error
SNV	Singular Normal Variate
TVC	Total Viable Counts
