# Supplementary material for: Fusion vs. Isolation: Evaluating the Performance of Multi-Sensor Integration for Meat Spoilage Prediction
Source: Foods. 2025 May 2;14(9):1613. doi: 10.3390/foods14091613 (PMC12071527; doi:10.3390/foods14091613)
Supplement: Supplementary file 1 [file foods-14-01613-s001.zip › foods-3592213-supplementary.pdf]

Supplementary Materials

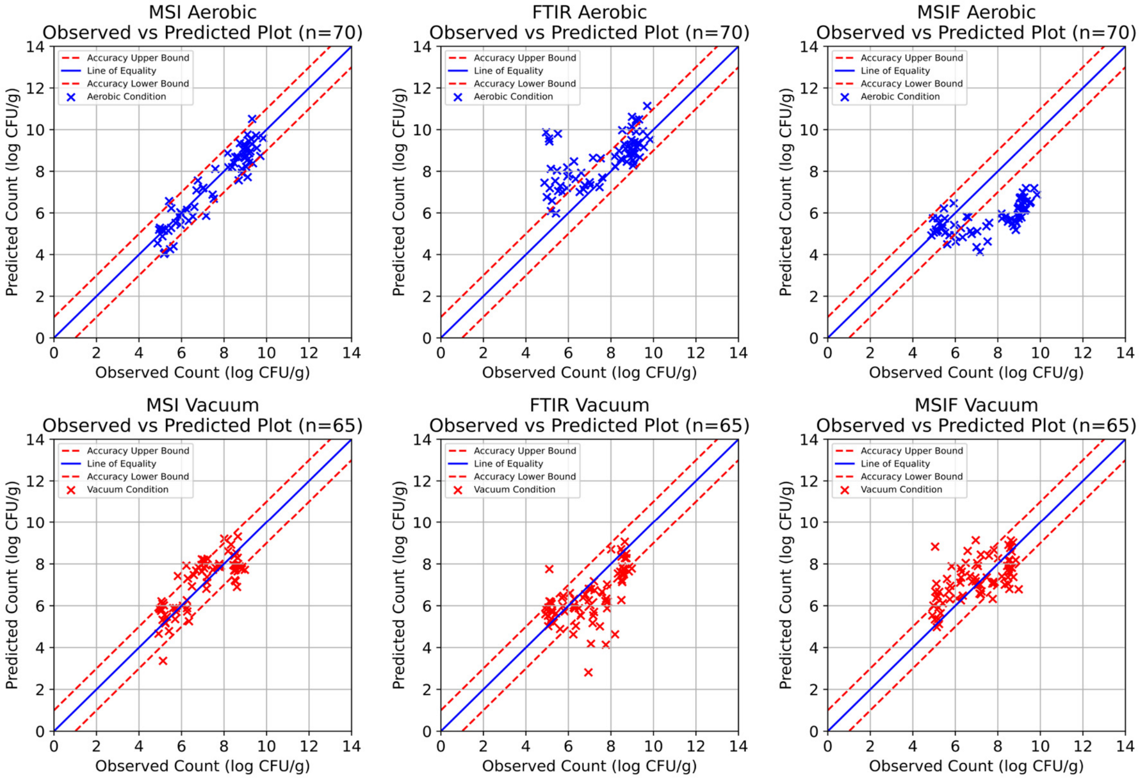

Supplementary Figure S1. Test performance plots for single-sensor, single-packaging-condition chicken thigh spoilage prediction models built using MSI, FTIR and MSIF datasets. MSI: Multi Spectral Imaging. MSIF: Multispectral Imaging Fluorescent. FTIR: Fourier Transform Infrared Spectroscopy (FTIR).

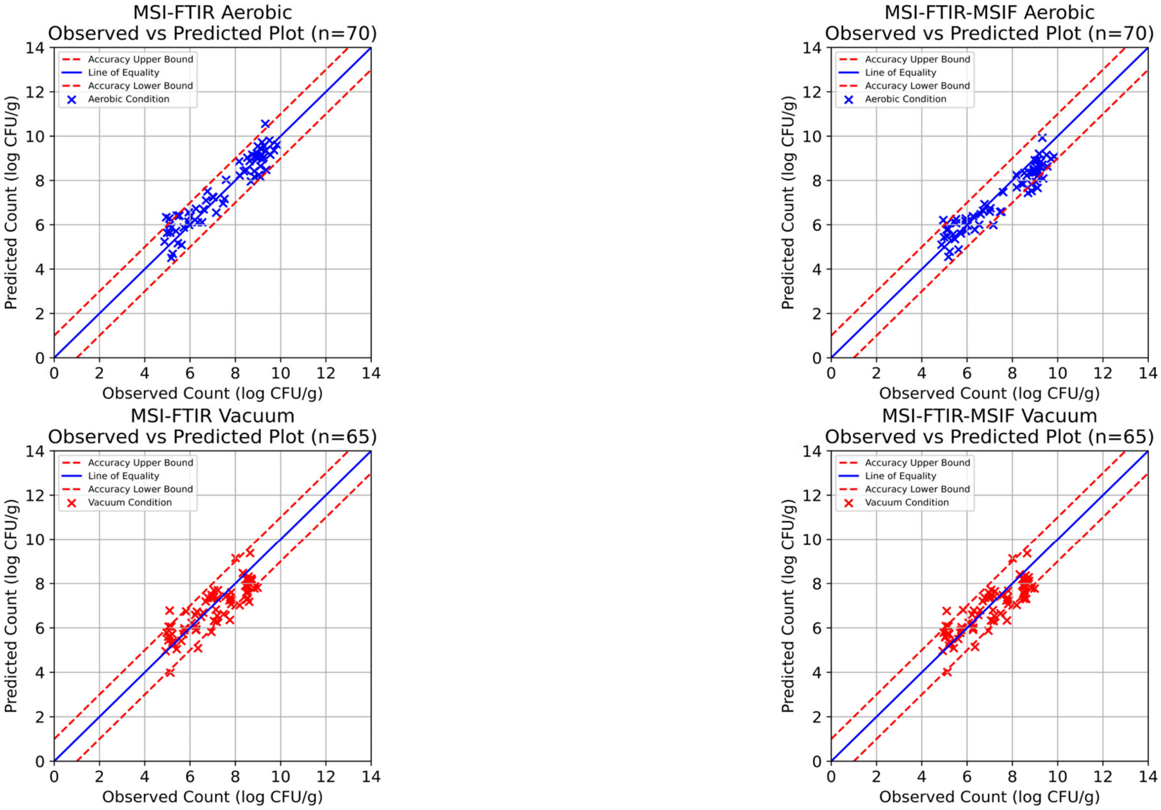

Supplementary Figure S2. Test performance plots for two/three-sensor, single-packaging-condition chicken thigh spoilage prediction models built using MSI, FTIR and MSIF datasets, built using the late (decision) fusion approach. MSI: Multi Spectral Imaging. MSIF: Multispectral Imaging Fluorescent. FTIR: Fourier Transform Infrared Spectroscopy (FTIR).

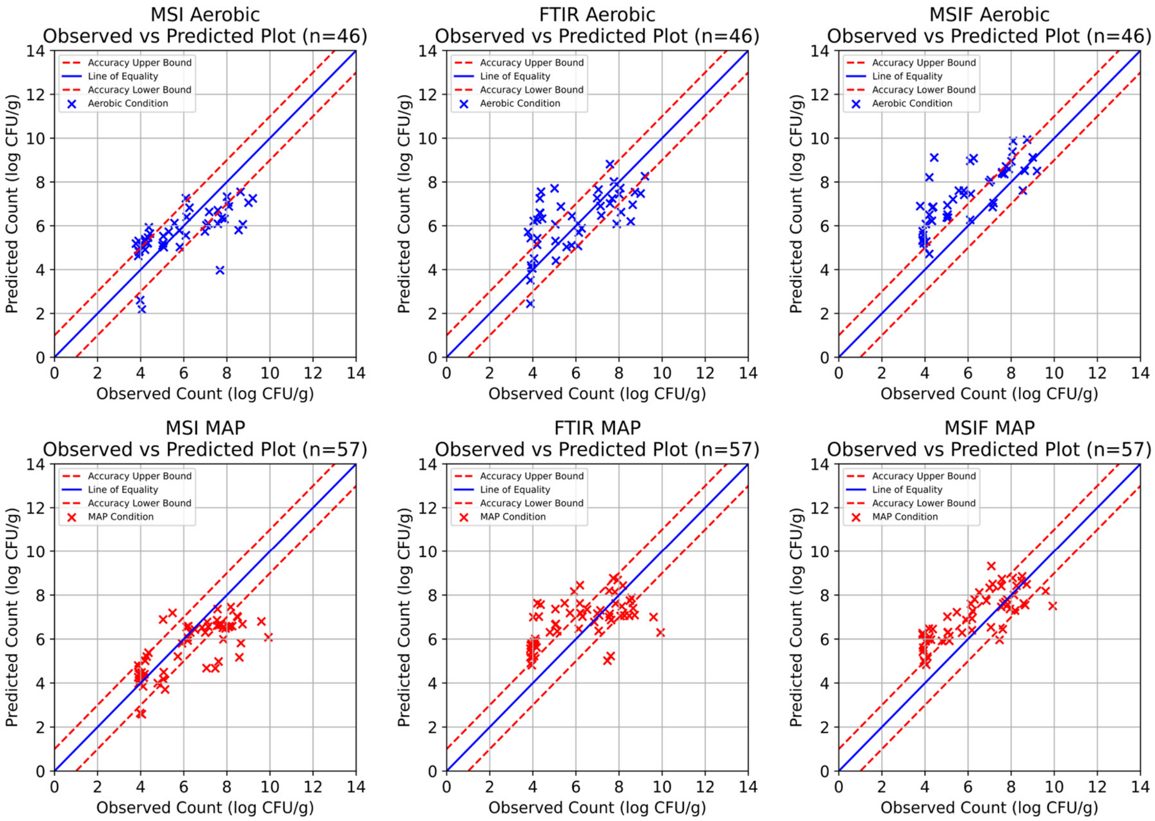

Supplementary Figure S3. Test performance plots for single-sensor, single-packaging-condition beef mince spoilage prediction models built using MSI, FTIR and MSIF datasets. MSI: Multi Spectral Imaging. MSIF: Multispectral Imaging Fluorescent. FTIR: Fourier Transform Infrared Spectroscopy (FTIR).

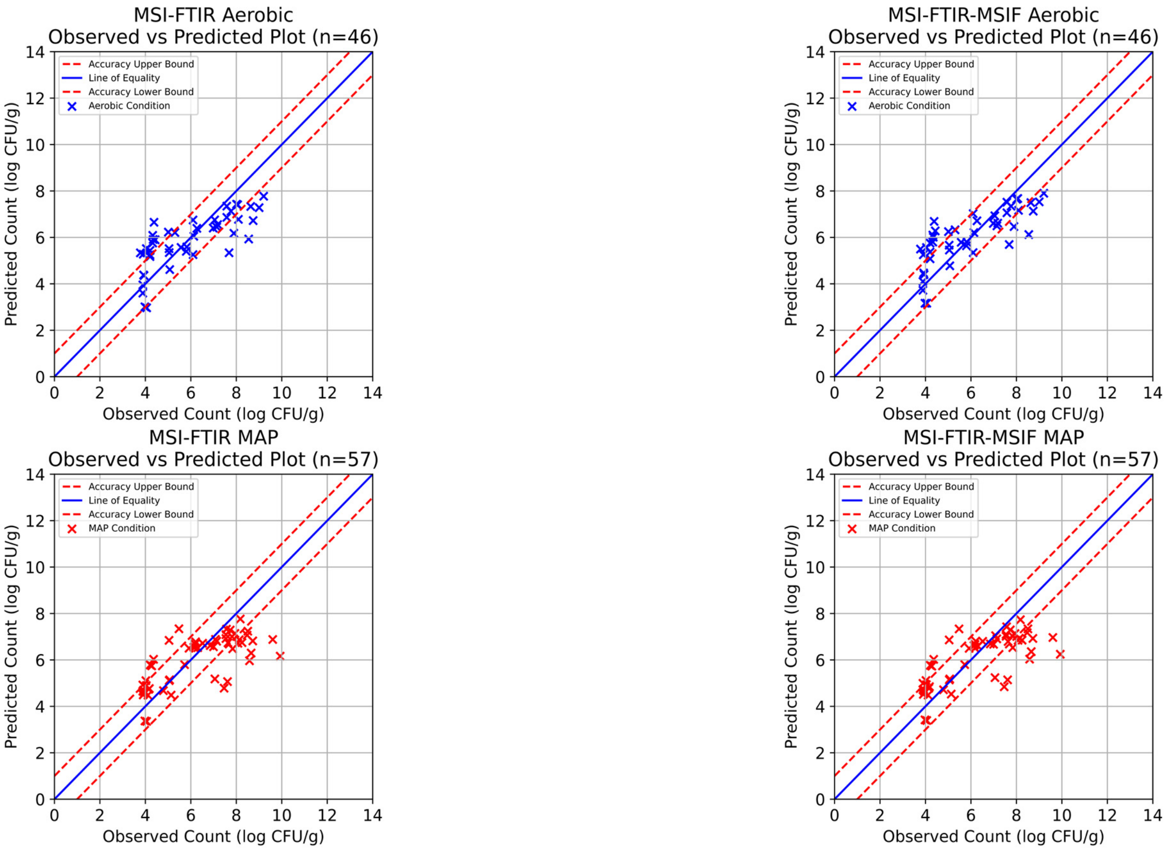

Supplementary Figure S4. Test performance plots for two/three-sensor, single-packaging-condition beef mince spoilage prediction models built using MSI, FTIR and MSIF datasets, built using the late (decision) fusion approach. MSI: Multi Spectral Imaging. MSIF: Multispectral Imaging Fluorescent. FTIR: Fourier Transform Infrared Spectroscopy (FTIR).

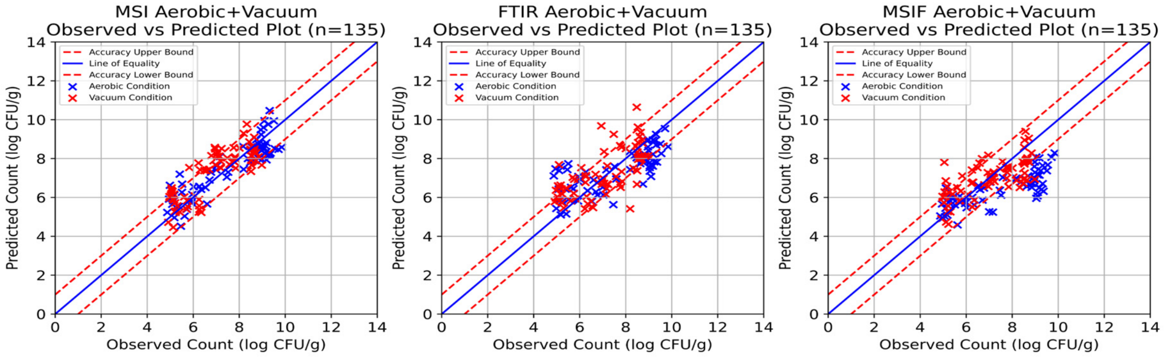

Supplementary Figure S5. Test performance plots for single-sensor, two-packaging-condition chicken thigh spoilage prediction models built using MSI, FTIR and MSIF datasets.

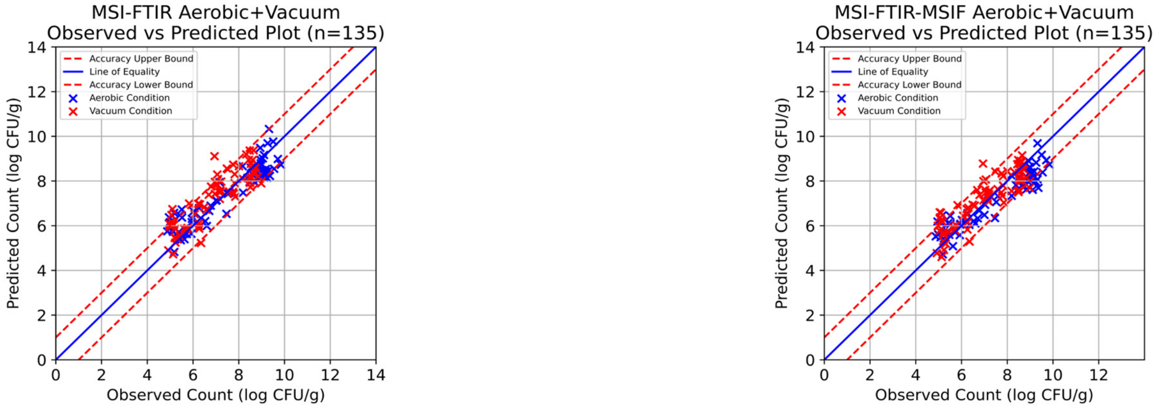

Supplementary Figure S6. Test performance plots for two/three-sensor, two-packaging-condition chicken thigh spoilage prediction models built using MSI, FTIR and MSIF datasets, built using the late (decision) fusion approach. MSI: Multi Spectral Imaging. MSIF: Multispectral Imaging Fluorescent. FTIR: Fourier Transform Infrared Spectroscopy (FTIR).

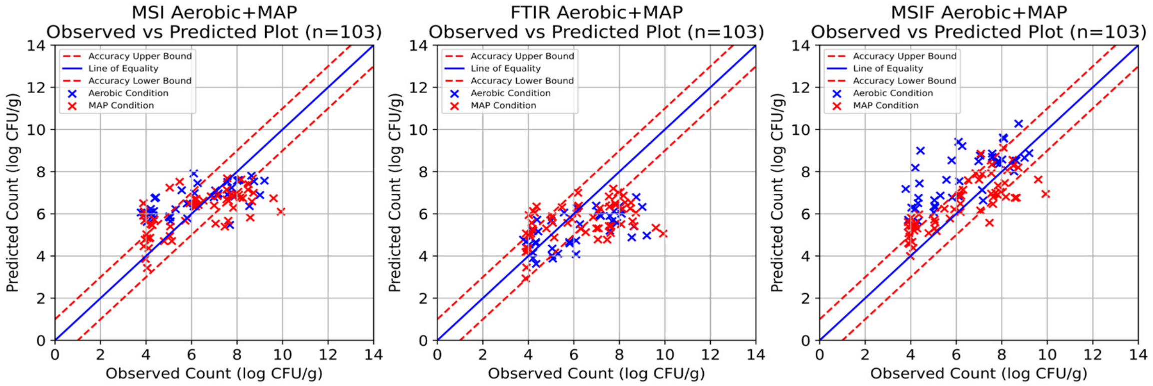

Supplementary Figure S7. Test performance plots for single-sensor, two-packaging-condition beef mince spoilage prediction models built using MSI, FTIR and MSIF datasets.

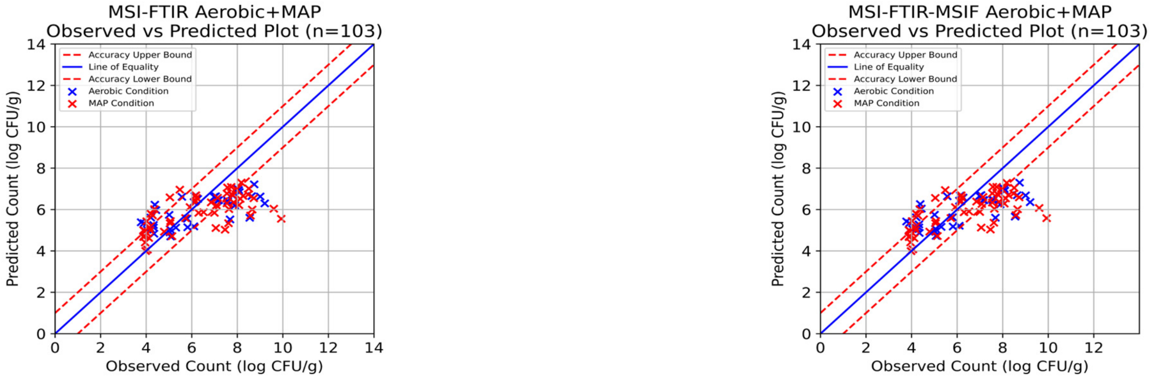

Supplementary Figure S8. Test performance plots for two/three-sensor, two-packaging-condition beef mince spoilage prediction models built using MSI, FTIR and MSIF datasets, built using the late (decision) fusion approach. MSI: Multi Spectral Imaging. MSIF: Multispectral Imaging Fluorescent. FTIR: Fourier Transform Infrared Spectroscopy (FTIR).
